# Intelligence, &c.

**Published:** 1827-07

**Authors:** 


### INTELLIGENCE, CORRESPONDENCE, &c.

### *Clinical Assistants.*

Our readers are aware that, for several years past, we have urged the propriety of selecting well qualified pupils for the registry of all interesting cases in our public hospitals—a practice so general on the Continent, and especially in Paris. We are glad to observe that this plan is about to be put into execution in the Westminster Hospital, and we hope it will soon be followed by all the great hospitals of town and country. It appears that the situation (which is for six months only) is to be a bonus or reward for merit. The individual is to be selected from the most deserving and best qualified pupils —is to undergo an examination—and then is to have board and lodging free of expence. We beg to suggest that, in order to make these institutions as useful as possible to the profession and the public, the pupil thus distinguished should not only be responsible for an accurate clinical history of the hospital during his sojourn, but should be expected to publish a clinical report for the period, in some of the medical journals. Without this, the plan will be confined to individual advantage, and the public welfare neglected. But there will not be wanting sufficient stimulus to the publication of such reports, in the notoriety and reputation thereby acquired by the pupil at the outset of his professional career. We hope to materially further this excellent plan by the prizes which we shall regularly confer on the best hospital reports.

P.S. We have had much pleasure in learning that the above post of honor, in the Westminster Hospital, has been conferred on Mr. Smith, who obtained the prize for the best hospital report in our last number.

---

*Incisions in Erysipelas.*

We understand that some observations which we made on this subject, in our last number, p. 537, have been misconstrued into an insinuation, that Mr. Copland Hutchison had been anticipated in the treatment of erysipelas phlegmonodes by incisions. We never meant any such insinuation, and have always distinctly named him as the first to employ this measure. The allusion to incisions in erysipelas, on board a line of battle ship in Basque roads, in the year 1809 or 1810, does not convey any such idea as has been entertained; for we know that Mr. Hutchison employed this remedy before those years, in the Royal Hospital at Deal.

---

We have received several communications, on the condition of being inserted *entire*, and as original papers. We beg to state, in expressing our thanks to those who have favored us, that we cannot break through the rule which we have made, to keep this work entirely for the purpose of analytical reviews of books, and Periscope of journals. All original papers must be published in the Extra-Limites department, and at the expense of the respective authors.

---

To Mr. Kingsley, and several of our Irish friends, we beg to say that their communications were forwarded to the monthly journals, and we see their receipt acknowledged there. But we cannot hold ourselves accountable for the subsequent disposal of the papers.

---

We were unable to give insertion to the interesting paper of Mr. T. G. of K——, on the use of ergot of rye in tedious labours. If he will permit its insertion in the Medical and Physical Jounal, or Repository, in the course of the quarter, we will take full notice of it in our next Periscope. Silence till the 20th of July will be considered *consent*.

---

*Internal Variolous Pustules.*
(Val de Grace.)

Doubts have been entertained of variolous pustules affecting the internal surfaces. The following case occurring in a public hospital, puts the matter, we think, beyond a doubt.

*Case.* Francis Lecoudre, aged 22 years, a private in the 39th regiment, entered the Val de Grace Hospital on the 16th July, having been ill eight days. On the 17th, in the morning, he presented the following phenomena:—he had been very delirious in the night—his eyes now haggard, and his face pale—tongue dry and brown—some spasms of the upper extremities—heat of surface intense—difficulty of swallowing—speech slow, but distinct—pulse small and above 100—coughs much. Leeches were immediately applied to the epigastrium, larynx, and temples, from the bites of which the blood flowed copiously. Lemonade *ad libitum*. 18th. The symptoms were somewhat mitigated. 19th. The patient is worse in all respects—face hippocratic. Sinapisms to the feet. 20th. The lower jaw is locked—convulsive movements in the limbs and trunk. He died on the 21st.

*Dissection.* The sinuses of the dura mater gorged with blood—brain firm—ventricles filled with sanguinolent serum—signs of inflammation in the pia mater and arachnoid. The lungs were sound, but the mucous membrane of the trachea and bronchia was of a vivid red colour, and the tubes filled with sanguinolent mucosities. No other diseased appearance in the chest. In the abdomen, the mucous membrane of the stomach and intestines, from the termination of the œsophagus to the rectum was covered with a pustular eruption, the eruptions being round, depressed in their centres, and filled with a limpid yellowish fluid. Several of the pustules were confluent, and all of them had exactly the appearance of small-pox pustules. On the most careful examination, no mark of variola, or of variolous vaccine inoculation could be detected on the surface of the body. The appearances on dissection, and the symptoms during the life of the patient, induced the physician (Dr. Damiron) to conclude, that it is possible, during an epidemic prevalence of variola, for the disease to affect the internal surface of the body alone—and that the above was a case of this kind.

---

*Stomach-Pump.*

Mr. Fox, House-Surgeon of the Derbyshire General Infirmary, has constructed a pump, adapted either for the stomach, or for the common purposes of an injecting syringe, and which we have seen. The simplicity of the construction will, we think, be a great recommendation to the instrument. The description and plates of the pump may be seen in the April Number of the Medical and Physical Journal, to which we refer our readers for an accurate idea of the instrument, which, we have no doubt, will soon be in all the shops for sale.

---

*Anatomical Persecution.*

The profession must have learnt with regret and indignation, the prosecution, or rather persecution, of Mr. Cooke, of Exeter, for disinterring and examining a body in that city. The law-suits instituted against him must have cost him a very large sum of money, besides the loss sustained in his professional pursuits by the revengeful, illiberal, and, we had almost said diabolical *mode* in which the prosecution was carried on. We think the Profession should, at once, shew their sympathy for Mr. Cooke, and their detestation of the course pursued by his persecutors, by a liberal subscription, not merely to reimburse the common law-expenses, but to assist him in re-establishing his practice. On such occasions as the present, the way to evince the feeling of the Profession, is by *small* but *numerous* contributions. With the view of aiding in this professional feeling, by adding to the impulse which has already been given, we have contributed our mite, and directed our publisher (Mr. Highley, 174, Fleet-street) to receive the names and contributions of our brethren, for the security and proper application of which, we will be answerable.

The names of the contributors will appear in our next number.—*June* 17, 1827.

P. S. After the above had gone to press we applied to Sir Astley Cooper, Mr. Brodie, and some other eminent individuals, for their names as the commencement or nucleus of a subscription. We learnt that, the very evening before, a meeting had taken place, and a *private* subscription had been set on foot, for the purpose of defraying the law expenses incurred by Mr. Cooke. We contributed our mite through that channel; but, with all due deference and respect for the opinions of the distinguished personages who have adopted the plan of a *private* subscription, we beg to state our entire dissent from such a proceeding. A private subscription befits a case of private distress—whereas a *public* grievance, oppression, or persecution, demands a *public* expression of sympathy for the aggrieved, and indignation against the aggression. In the publicity of this sympathy and feeling consists the main *levamen* of the injury sustained by the individual—and not in the mere reimbursement of a certain number of pounds expended in law. We therefore persevere in recommending a *public* subscription, however small the contribution, and we pledge ourselves to publish the names (the amount of individual contribution need not be published) in our next number. Mr. Highley, will receive all names and subscriptions for that purpose from the 1st July, till the 15th September.

The public and Mr. Cooke have now the satisfaction of knowing that the first professional characters in this metropolis *have* unanimously agreed to forward a subscription—and we feel no compunction of conscience in giving *publicity* to this fact, even against the consent of the parties concerned.

---

We are unable to give insertion to the letter of Dr. Brown, of Preston, respecting his mode of treating intermittent fever, for the reasons already assigned, that this is *not* a journal for *original communications*. Were we to give insertion to all the letters that come to us, respecting subjects animadverted on in this and in other journals, we should not have a single page left for the proper and avowed objects of the work. The EXTRA-LIMITES department is open for all those who defray the expense of paper and type. In this department, a sacrifice is made by individuals to the public good. We make a considerable sacrifice ourselves, in the *extra quantity* of letter-press which we give in this journal; but we cannot carry that beyond certain limits.

---

*Medico-Chirurgical Society.*

We are happy to be able to state, that our anticipations last quarter have been amply realized. The Society is, at this moment, in a more flourishing condition than at any former period since its first establishment. Papers are crowding in from the highest orders of the Profession, and the applications for admission, as members, are more numerous than ever they were. The council has been obliged to call extra meetings every second Tuesday throughout the last month, in order to get through the numerous and important papers transmitted to the Society. A volume of Transactions will now be regularly published every year—a circumstance that will induce many to contribute papers, who would otherwise hold back, from the uncertainty of publication. Some very valuable papers have lately been read, from Messrs. Lawrence, Stanley, Earle, and other distinguished surgeons of this metropolis.

Dr. Clark has in the press, an Essay on the Effects of Climate on Disease, including Directions to Invalids going abroad for the recovery of their Health. To which is added, some account of the principal Mineral Waters of the Continent, and of their Effects in Chronic Diseases.

After our last number was closed, we received a letter from Mr. Lambert, calling on us for explanation relative to an observation made on an *anonymous* report from Guy's Hospital, and holding out a *personal threat*, if we did not do so. Mature reflexion has led us to refuse insertion to his letter—for three reasons, *first*, because no observations were made on Mr. Lambert, and no man has a right to call retrospectively for explanations respecting an *anonymous* paper—*secondly*, because a threat was held out in the letter, (and personal threats we shall ever despise)—*thirdly*, because the language in which Mr. Lambert's note is couched, is totally inadmissible.

### *Emaciation of the Heart.*

This disease, though not nearly so frequent as the opposite extreme, hypertrophy, is yet by no means rare. The following case is interesting.

A young lady, 20 years of age, of sound constitution and good health, was attacked in March, 1826, with a catarrhal disease, which prevailed in Austria at that time. On the sixth day there was a considerable aggravation of the complaint, the cough being dry, with pain in the thorax. She could not bear the least noise, and had intense cephalalgia, without fever. In the evening she had a slight rigor succeeded by heat, after which she fell into a slumber and awoke in a fright, complaining of oppression and sense of dying. Ten ounces of blood were taken from her arm, but the sense of oppression and other distressing feelings were increased by the loss of blood. The breathing became short, the pulse irregular, the pulsations of the heart and carotids violent, the hands and feet cold. "When the hand was applied to the chest there was felt an agitation and noise resembling that of boiling water." The intellectual faculties were tranquil. To these symptoms were added vomiting and purging. Towards evening there was some mitigation of her sufferings, which was only a prelude to death, which took place at eight o'clock, in the full possession of her mental powers.

The body was examined 17 hours after death, when the lungs were found gorged with blood, and the bronchia filled with sero-sanguinolent frothy fluid. There was some effusion in the right side of the chest. But the most remarkable phenomenon was the wasting of the heart. The parietes of the right ventricle were not thicker than the back of the scalpel—those of the left side about three times that of the right. The muscular structure was quite rotten, and easily meshed between the fingers. The size of the heart generally was not more or less than natural.

The above case offers an example, not very rare, of a vital organ being in a very bad state, yet without any adequate symptoms occurring till some accidental commotion in the system, when the diseased organ quickly fails in its function, and death is the consequence.

As a very remarkable contrast to the above case, we this day examined the body of a child, six months old, who died rather suddenly and unexpectedly, while attended by Mr. Fincham, of Spring Gardens. Every part of the body was in a state of perfect integrity, except the heart. The parietes of the left ventricle were more than half an inch in thickness—in fact, they were as thick as those of an adult heart. The cavity of the ventricle was so small that it would not contain half a tea-spoonful of fluid. This was the most remarkable specimen of hypertrophy of the ventricular parietes we have ever seen. Yet this infant exhibited no particular symptoms of disorder till within a day or two of its death, when its breathing became disturbed, and, as was mentioned before, it suddenly died.

